# Embolization of the internal maxillary artery via open retrograde superficial temporal artery approach

**DOI:** 10.1093/jscr/rjab508

**Published:** 2021-11-12

**Authors:** Lauren E Miller, Suresh Mohan, Walid I Essayed, Madhav Sukumaran, Shervin Mirshahi, James D Rabinov, Rose Du, Eleni M Rettig

**Affiliations:** Department of Otolaryngology – Head and Neck Surgery, Massachusetts Eye and Ear Infirmary, Boston, MA, USA; Division of Otolaryngology – Head and Neck Surgery, Department of Surgery, Brigham and Women’s Hospital, Boston, MA, USA; Department of Otolaryngology – Head and Neck Surgery, Massachusetts Eye and Ear Infirmary, Boston, MA, USA; Division of Otolaryngology – Head and Neck Surgery, Department of Surgery, Brigham and Women’s Hospital, Boston, MA, USA; Department of Neurosurgery, Brigham and Women’s Hospital, Boston, MA, USA; Department of Neurosurgery, Brigham and Women’s Hospital, Boston, MA, USA; Department of Radiology, Brigham and Women’s Hospital, Boston, MA, USA; Departments of Neurosurgery and Radiology, Massachusetts General Hospital, Boston, MA, USA; Department of Neurosurgery, Brigham and Women’s Hospital, Boston, MA, USA; Division of Otolaryngology – Head and Neck Surgery, Department of Surgery, Brigham and Women’s Hospital, Boston, MA, USA

## Abstract

Sentinel bleeds in head and neck cancer patients present as an ominous symptom often necessitating urgent endovascular embolization. However, this approach can be complicated in patients who have previously undergone head and neck cancer ablation and reconstruction, thus altering the standard arterial vascular supply. Herein we describe an innovative method of internal maxillary artery (IMA) access in a patient with a sentinel bleed who previously underwent proximal external carotid artery (ECA) rerouting for free flap reconstruction. The open retrograde superficial temporal artery approach for IMA embolization is minimally invasive and effective and should be considered for head and neck cancer patients at risk of hemorrhage from distal ECA branches without a proximal ECA embolization option.

## INTRODUCTION

Sentinel bleeds in head and neck cancer patients present as an ominous and urgent symptom that can occur due to vascular complications such as pseudoaneurysm, thrombosis or arteriovenous fistula following prior surgical manipulation, tumor progression or chemoradiation [1, 2]. The preferred treatment option in these cases is often endovascular embolization. However, this approach can be complicated in patients who have previously undergone head and neck cancer ablation and reconstruction, thus altering the standard arterial vascular supply. Herein, we describe an innovative method for obtaining access to the internal maxillary artery (IMA) through an open retrograde approach via the superficial temporal artery (STA) after proximal sacrifice of the external carotid artery (ECA) in a head and neck cancer patient.

## CASE REPORT

A 43-year-old male underwent tooth extraction for jaw pain and experienced heavy bleeding; prompting imaging that revealed a large mass of the left maxilla, invading the hard palate and orbital floor. He underwent prophylactic endovascular coil embolization of his left IMA prior to biopsy of the mass to prevent further hemorrhage. He was diagnosed with a solid type adenoid cystic carcinoma of the left maxilla, T4N0M0. He was referred to Otolaryngology-Head and Neck Surgery and underwent hemimaxillectomy with a three-segment stacked fibula free flap reconstruction guided by preoperative virtual surgical planning. The vascular pedicle did not have sufficient length to anastomose to branches of the external carotid artery, so a vein graft was used to create a Corlett loop reaching from the neck to the reconstructive site. Initial attempts to anastomose to the facial artery were complicated by thrombosis; thus, the distal external carotid artery was used and the flap successfully revascularized. The patient recovered uneventfully and underwent adjuvant proton irradiation with concurrent chemotherapy. Following the completion of radiation, he developed dehiscence of the posterior extent of the reconstructed palate that was successfully managed with a palatal obturator.

**Figure 1:**
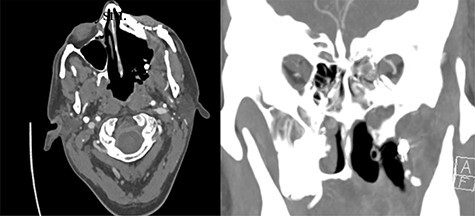
Evidence of partially extruded embolization material within the left infratemporal fossa along the left maxillectomy defect in axial (left) and coronal (right) sections. Coronal view also demonstrates more inferior contrast blush concerning for pseudoaneurysm.

Eight months after treatment completion, the patient removed a small wire from his maxillectomy cavity that was consistent with a vascular coil from his previous IMA embolization. CT angiography was obtained notable for partial coil extrusion from a left IMA branch, with a separate vascular blush concerning for pseudoaneurysm of another IMA branch (Fig. 1). Several days later, he developed left-sided epistaxis, which was controlled with nasal packing and subsided after approximately 30 minutes. This was felt to represent a possible sentinel bleed; although his ECA was ligated proximally, there was thought to be sufficient backflow to fill the pseudoaneurysm and cause symptomatic bleeding, as has been reported previously [3].

Due to inability to access patient’s IMA via the ECA as it had previously been utilized for free flap reconstruction, options considered included endoscopic exploration of the maxillectomy cavity for direct vessel ligation; neck exploration or percutaneous approach to access the distal stump of the ECA; or retrograde access for IMA endovascular embolization via the undissected STA. The decision was made to attempt retrograde access via the STA.

**Figure 2:**
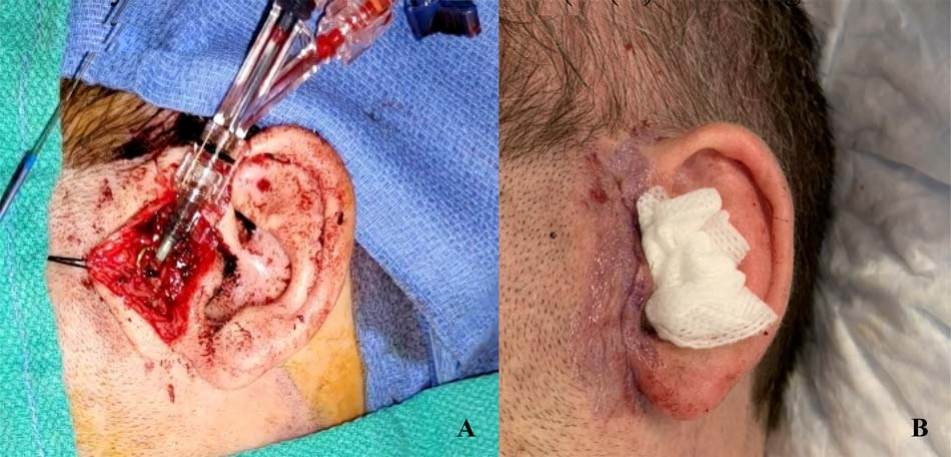
Cannulation of the STA through preauricular incision (**A**). Multilayered closure with superficial glue following decannulation (**B**).

**Figure 3:**
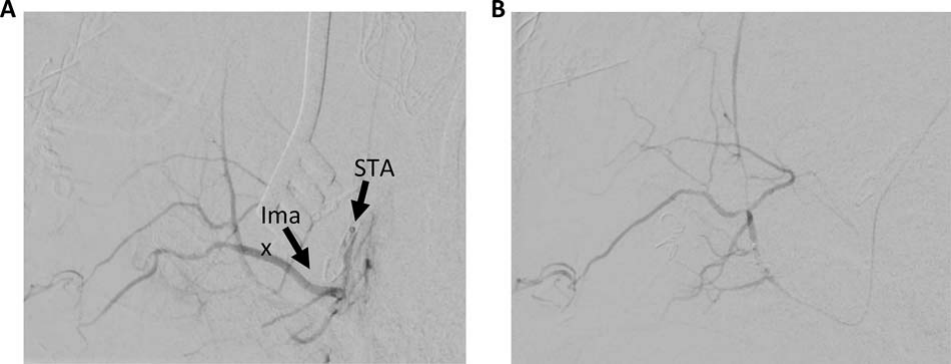
Left STA injection. (**A**) Pre-embolization and (**B**) post-embolization. Ima, internal maxillary artery.

The patient underwent a left preauricular incision to isolate the STA from surrounding parotid parenchyma by the Head & Neck Surgery team. Under microscopic guidance, the STA was cannulated with an 18-gauge angiocatheter (Fig. 2) by the Neurosurgery team, which was secured in place with 2-0 silk suture. A rotating hemostatic valve (Abbott Laboratories, Chicago, IL) was attached to the angiocatheter and a retrograde angiogram was performed, confirming STA access (Fig. 3A). An Echelon 10 microcatheter (Medtronic, Minneapolis, MN) and a Synchro 14 microwire (Stryker, Kalamazoo, MI) were then used to select the proximal left IMA for digital biplane angiography. Hand-injected control angiography confirmed appropriate microcatheter positioning, demonstrating opacification of the left IMA and branches, with nonspecific contrast blush including the left sphenopalatine arteries. Thereafter, liquid embolization using Onyx® Liquid Embolic System (Medtronic, Minneapolis, MN) was performed to occlude the IMA. A final angiogram demonstrated satisfactory occlusion of the left IMA without evidence of extravasation, aneurysm or pseudoaneurysm (Fig. 3B). The STA was decannulated and ligated, and the preauricular incision was closed primarily.

The patient tolerated the procedure well and was discharged on postoperative day 1 in stable condition. In follow-up on postoperative day 10, he had no further evidence of epistaxis or other symptoms.

## DISCUSSION

Endovascular embolization techniques offer an efficient and successful option for managing urgent bleeding difficult to access otherwise. However, in head and neck cancer patients, vascular anatomy is often altered in the context of prior ablative and reconstructive surgery, posing significant challenges to standard approaches for arterial embolization. We describe an innovative method of IMA access in a patient who previously underwent proximal ECA rerouting for free flap reconstruction. As free flap reconstruction is increasingly standard of care for the complex defects created by head and neck cancer ablation, this technique is a useful alternative to access branches of the distal ECA when it has been previously ligated or rerouted proximally.

There is one previous similar report, to our knowledge, of open retrograde STA access to the IMA, for successful embolization using polyvinyl alcohol particles of an arteriovenous malformation with persistent hemorrhage after previous ECA embolization [3]. Another case series described percutaneous puncture of the distal ECA and its branches, includes three cases of percutaneous STA catheterization, although notably this technique was associated with a high complication rate of hematoma at the puncture site and inadvertent internal carotid artery puncture [4]. In our case, the open approach to the STA was felt to be optimal given the ease of access with a small preauricular incision, the advantages of manual manipulation given the small size and tortuosity of the STA and challenging location of the distal stump of the ECA deep to the mandible in an operated and radiated field.

## CONCLUSION

The open retrograde STA approach for IMA embolization is minimally invasive and effective and should be considered for head and neck cancer patients at risk of hemorrhage from distal ECA branches without a proximal ECA embolization option.
